# Inhibition of NF-κB by deoxycholic acid induces miR-21/PDCD4-dependent hepatocellular apoptosis

**DOI:** 10.1038/srep17528

**Published:** 2015-12-01

**Authors:** Pedro M. Rodrigues, Marta B. Afonso, André L. Simão, Pedro M. Borralho, Cecília M. P. Rodrigues, Rui E. Castro

**Affiliations:** 1 Research Institute for Medicines (iMed.ULisboa), Faculty of Pharmacy, Universidade de Lisboa, 1649-003 Lisboa, Portugal; 2 Department of Biochemistry and Human Biology, Faculty of Pharmacy, Universidade de Lisboa, 1649-003 Lisboa, Portugal

## Abstract

MicroRNAs (miRNAs/miRs) are key regulators of liver metabolism, while toxic bile acids participate in the development of several liver diseases. We previously demonstrated that deoxycholic acid (DCA), a cytotoxic bile acid implicated in the pathogenesis of non-alcoholic fatty liver disease, inhibits miR-21 expression in hepatocytes. Here, we investigated the mechanisms by which DCA modulates miR-21 and whether miR-21 contributes for DCA-induced cytotoxicity. DCA inhibited miR-21 expression in primary rat hepatocytes in a dose-dependent manner, and increased miR-21 pro-apoptotic target programmed cell death 4 (PDCD4) and apoptosis. Both miR-21 overexpression and PDCD4 silencing hampered DCA-induced cell death. Further, DCA decreased NF-κB activity, shown to represent an upstream mechanism leading to modulation of the miR-21/PDCD4 pathway. In fact, NF-κB overexpression or constitutive activation halted miR-21-dependent apoptosis by DCA while opposite results were observed upon NF-κB inhibition. In turn, DCA-induced oxidative stress resulted in caspase-2 activation and NF-κB/miR-21 inhibition, in a PIDD-dependent manner. Finally, modulation of the NF-κB/miR-21/PDCD4 pro-apoptotic pathway by DCA was also shown to occur in the rat liver *in vivo*. These signalling circuits may constitute appealing targets for bile acid-associated liver pathologies.

Toxic bile acids act as potent inducers of hepatocyte apoptosis and contribute to the pathogenesis of several liver diseases^1^. In particular, deoxycholic acid (DCA) is found increased in the serum of non-alcoholic steatohepatitis (NASH) patients^2^ and contributes to the development of hepatocellular carcinoma (HCC)^3^. The cytotoxic effects of DCA are largely attributed to its ability to induce apoptosis^4^,^5^. DCA-induced apoptosis may be triggered through activation of the extrinsic apoptotic pathway, with Fas receptor and death receptor 5 acting as key players^6^,^7^. In parallel, DCA also directly triggers the intrinsic pathway of apoptosis, by decreasing inner mitochondrial transmembrane potential and enhancing levels of reactive oxygen species (ROS). This results in mitochondrial permeabilization and consequent release of cytochrome *c* and Smac/DIABLO^8^,^9^. Finally, the proapoptotic stress-activated kinase c-Jun N-terminal kinase (JNK) is also activated by DCA^10^. In that regard, we have recently shown that JNK1/c-Jun activation of the p53/microRNA-34a/Sirtuin 1 pathway contributes to apoptosis induced by DCA in the rat liver^11^. Nevertheless, the exact mechanisms and signalling networks modulated by DCA during induction of hepatocyte cell death remain scattered.

microRNAs (miRNAs/miRs) are endogenous small non-coding RNAs that modulate gene expression in a post-transcriptional manner, inhibiting protein translation or inducing mRNA deadenylation and decay^12^,^13^. Deregulation of miR-21 associates with several liver diseases and its serum levels have been proposed as possible biomarkers for NASH, hepatitis C virus infection and HCC^14^. Programmed cell death 4 (PDCD4) and phosphatase and tensin homolog (PTEN) are the two best-studied and characterized miR-21 targets^15^. In the liver, miR-21 has been shown to regulate expression of PTEN in human HCC^16^, and to activate hepatic stellate cells *via* PTEN/Akt signalling^17^. In parallel, miR-21 overexpression in human cholangiocarcinoma inhibits PDCD4^18^, while promoting migration and invasion through PDCD4 and activator protein 1 (AP-1) in HCC^19^. More recently, the miR-21/PDCD4/AP-1 pathway was identified as a driving force for development of hepatic fibrosis^20^. The mechanisms by which PDCD4 induces apoptosis remain poorly resolved. Ultimately, it may lead to apoptosis by interfering with JNK-mediated c-Jun phosphorylation and inhibiting AP-1 transactivation^21^; activating cell cycle inhibitor p21 thus suppressing cyclin-dependent kinase 1^22^; and hampering protein translation by directly interacting with different translation initiation factors^23^,^24^.

We have previously demonstrated that DCA decreases miR-21 levels in primary rat hepatocytes^25^. In turn, both DCA and miR-21 have been implicated in the pathogenesis of liver diseases with high levels of cell death, like NASH. We aimed to elucidate the mechanisms by which DCA inhibits miR-21 and to evaluate whether these events are relevant for DCA-induced hepatocyte cell death. In particular, we sought to evaluate whether nuclear factor kappa Β (NF-κB), a transcription factor recently linked to miR-21^26^,^27^, may act as an upstream regulator of the miR-21 pathway by DCA, and to ascertain the contribution of oxidative stress in this signalling network.

## Results

### DCA inhibits the miR-21/PDCD4 pathway in primary rat hepatocytes in a time- and dose-dependent manner

DCA is a well-established inducer of hepatocyte cell death, through modulation of several apoptosis-related signalling pathways^5^,^6^,^9^,^11^,^28^. We have recently shown that ursodeoxycholic acid (UDCA), a cytoprotective and anti-apoptotic bile acid, shifts the liver miRNA expression pattern *in vivo* towards a proliferative environment, inducing miR-21 expression both during liver regeneration and in cultured HepG2 cells. On the other hand, DCA significantly inhibits miR-21 expression in primary rat hepatocytes^25^. Still, the relevance of DCA-mediated inhibition of miR-21 during hepatocellular injury, as well as the underlying mechanistic signalling events remains unknown.

**Figure 1:**
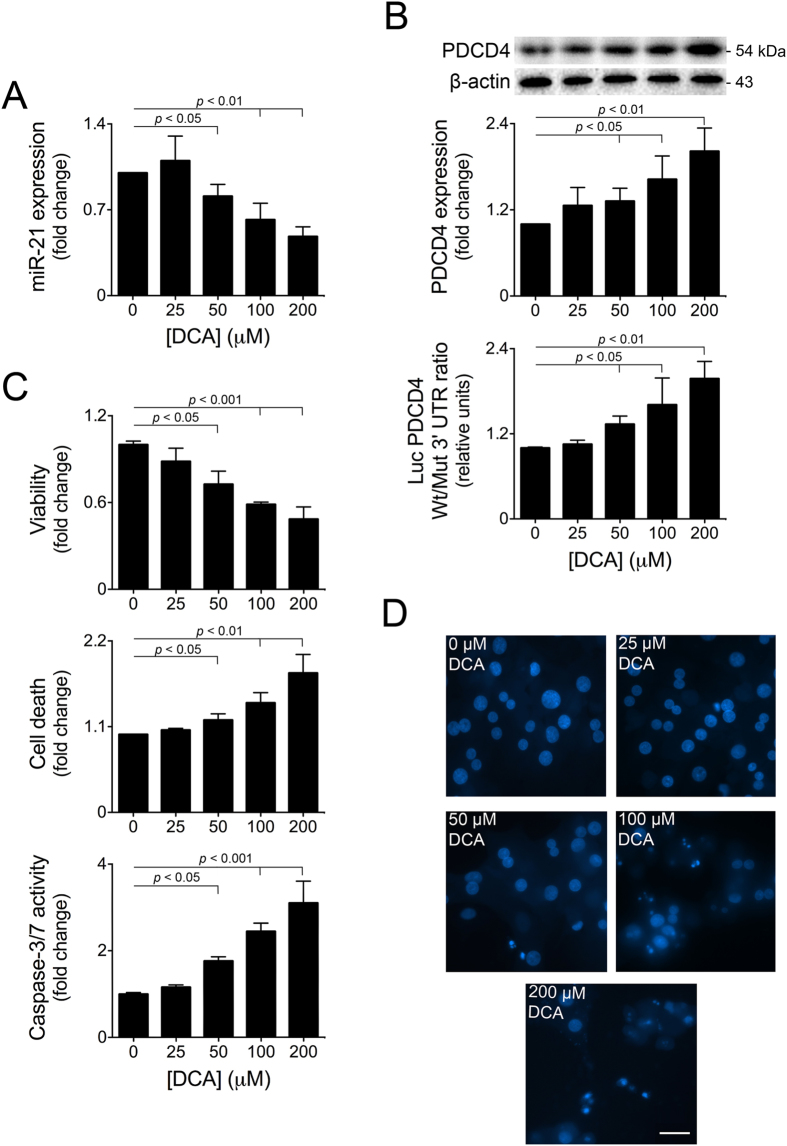
DCA inhibits miR-21 expression in primary rat hepatocytes in a dose-dependent manner. Hepatocytes were isolated and plated as described in Materials and Methods and treated with 25 to 200 μM DCA or no addition (control) for 24 h. (**A**) Real-Time RT-PCR analysis of miR-21 (n = 7). (**B**) Immunoblotting of PDCD4 (top; n = 7) and ratio between Wt and Mut miR-21 luciferase activity (bottom; n = 5). Representative blots are shown. Blots were normalized to endogenous β-actin. Cells were co-transfected with a reporter vector consisting of a luciferase cDNA fused to the 3′ UTR of PDCD4, containing either a Wt or Mut miR-21 binding site. The cytomegalovirus-*Renilla* luciferase vector was used as an internal standard control. (**C**) Cell viability, measured by the ApoTox-Glo^TM^ Triplex assay (top; n = 5), cell death measured by the LDH assay (middle; n = 7) and caspase-3/7 activity (bottom; n = 5). (**D**) Apoptotic cells were detected by Hoechst staining. Representative images of control and 25, 50, 100 and and 200 μM DCA are shown. Bar, 30 μM. Arrows indicate apoptotic nuclei. Results are expressed as mean ± SEM fold change.

We first evaluated whether inhibition of miR-21 by DCA occurs in a dose-dependent manner. Our results show that primary rat hepatocytes incubated with 50 to 200 μM DCA for 24h decreased miR-21 expression between 20 to ~50% (at least *p* < 0.05) (Fig. 1A), comparing with controls. In the liver, miR-21 is generally associated with proliferative/anti-apoptotic functions, through targeting of PTEN and PDCD4^16^,^29^,^30^,^31^. We next evaluated whether inhibition of miR-21 by DCA correlated with an increase in PTEN and/or PDCD4 protein levels. While PTEN expression was not significantly modulated (unpublished observations), DCA increased PDCD4 protein levels up to ~2-fold (at least *p* < 0.05) (Fig. 1B top). To evaluate whether DCA modulates PDCD4 expression *via* miR-21, cells were co-transfected with a luciferase reporter construct containing the wild-type miR-21 binding site within the PDCD4 3′UTR (Luc-PDCD4 Wt 3′UTR) or a mutated miR-21 binding site (Luc-PDCD4 Mut 3′UTR)^32^. In agreement with the previous results, PDCD4 luciferase activity increased up to 2-fold in cells incubated with >50 μM DCA (at least *p* < 0.05) (Fig. 1B bottom), suggesting that PDCD4 is modulated by DCA *via* miR-21.

In line with the miR-21 inhibitory pattern, concentrations of DCA > 50 μM significantly decreased cell viability between 30 to 50% (at least *p* < 0.05) (Fig. 1C top). Concomitantly, DCA-induced general cell death, as measured by LDH release, increased up to ~2-fold, in a dose-dependent manner (at least *p* < 0.05) (Fig. 1C middle). To assess whether apoptosis is the predominant form of cell death induced by this range of DCA concentrations, we measured activation of caspase-3 and visualized nuclear fragmentation and shrinking by Hoechst staining. Caspase-3 activation increased, in a dose-dependent manner, up to ~3-fold (*p* < 0.001 for >50 μM DCA) (Fig. 1C bottom). In agreement, cells incubated with DCA displayed increased apoptotic nuclei (Fig. 1D), particularly for the 100 and 200 μM DCA concentrations. Altogether, DCA-induced apoptosis is inversely correlated with miR-21 expression and positively correlated with PDCD4 expression pattern, suggesting that DCA-induced apoptosis occurs, at least in part, *via* miR-21/PDCD4. Of note, concentrations of DCA above 200 μM are less capable of modulating miR-21/PDCD4 and mostly induced necrosis (unpublished observations). These effects likely reflect the intrinsic detergent properties of DCA at high concentrations.

We also evaluated whether modulation of the miR-21/PDCD4 pathway by DCA is time-dependent (Figure S1). Cells were incubated with 100 μM DCA or no addition (control) for 4, 16, 24, 40 and 48 h. DCA inhibited miR-21 expression throughout time, starting at 16h of incubation (*p* < 0.05). In agreement, DCA-induced PDCD4 protein levels remained elevated in the 16–48 h timeframe (at least *p* < 0.05).

### DCA induces apoptosis of primary rat hepatocytes by engaging the miR-21/PDCD4 pathway

**Figure 2:**
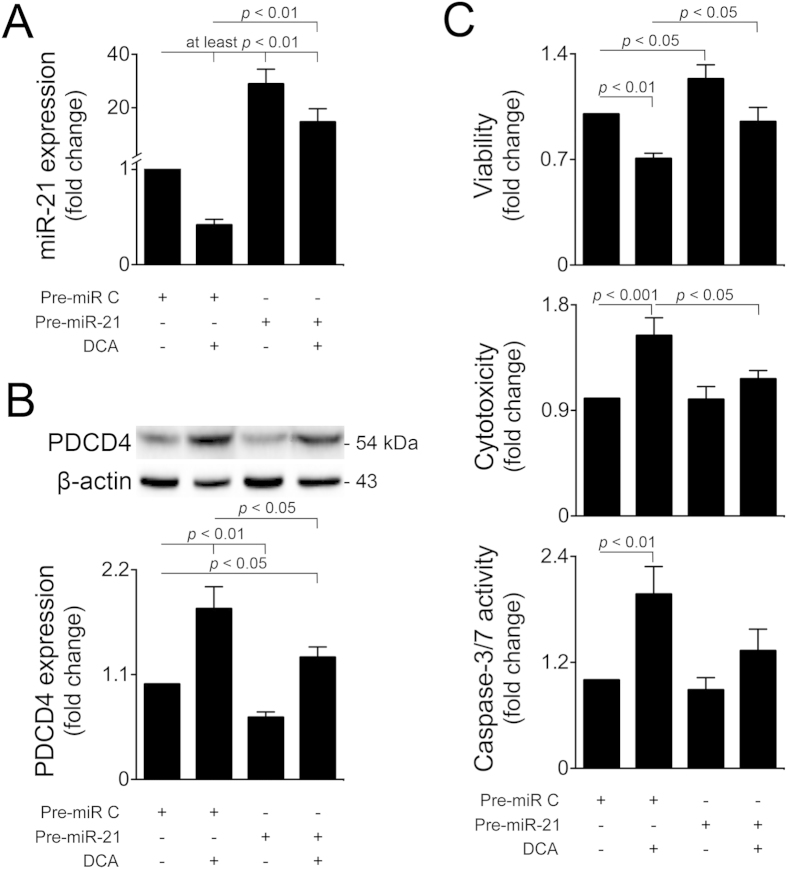
mir-21 overexpression counteracts DCA-induced apoptosis. Primary rat hepatocytes were transfected with a miR-21 precursor (Pre-miR-21) or control (Pre-miR-C) and treated with 100 μM DCA or no addition for 24 h. (**A**) Real-Time RT-PCR analysis of miR-21 expression (n = 7). (**B**) Immunoblotting of PDCD4 (n = 5). Representative blots are shown. Blots were normalized to endogenous β-actin. (**C**) Cell viability (top), cytotoxicity (middle) and caspase-3/7 activity (bottom) measured by the ApoToxGlo^TM^ Triplex assay (n = 5). Results are expressed as mean ± SEM fold change.

To evaluate the functional relevance of miR-21 during DCA-induced cell death, we transfected primary rat hepatocytes with a miR-21 precursor, both in the presence or absence of DCA. As expected, miR-21 was significantly overexpressed in cells transfected with Pre-miR-21 (*p* < 0.001), when compared to cells transfected with Pre-miR-Control (Fig. 2A). In Pre-miR-Control-transfected cells, DCA inhibited miR-21 expression by 60% (*p* < 0.01). DCA also counteracted miR-21 overexpression in Pre-miR-21-transfected cells, although to a lesser extent. miR-21 overexpression alone reduced PDCD4 levels by almost 40% (*p* < 0.01) (Fig. 2B). In these conditions, the ability of DCA to increase PDCD4 protein expression was significantly compromised, comparing with DCA-treated Pre-miR-Control cells. As previously, no significant changes were observed in PTEN protein levels following miR-21 overexpression (unpublished observations), further substantiating PDCD4 as a prime target modulated by DCA *via* miR-21 in primary rat hepatocytes. Surprisingly, although miR-21 overexpression alone significantly increased cell viability by ~20% (*p* < 0.05) (Fig. 2C top), effects on cytotoxicity (Fig. 2C middle) and caspase-3 activity (Fig. 2C bottom) were almost absent. Nevertheless, miR-21 overexpression almost completely abrogated DCA-mediated reduction in cellular viability; increase in cytotoxicity; and caspase-3 activation. These results further confirm that the miR-21/PDCD4 pathway mediates DCA-induced apoptosis.

**Figure 3:**
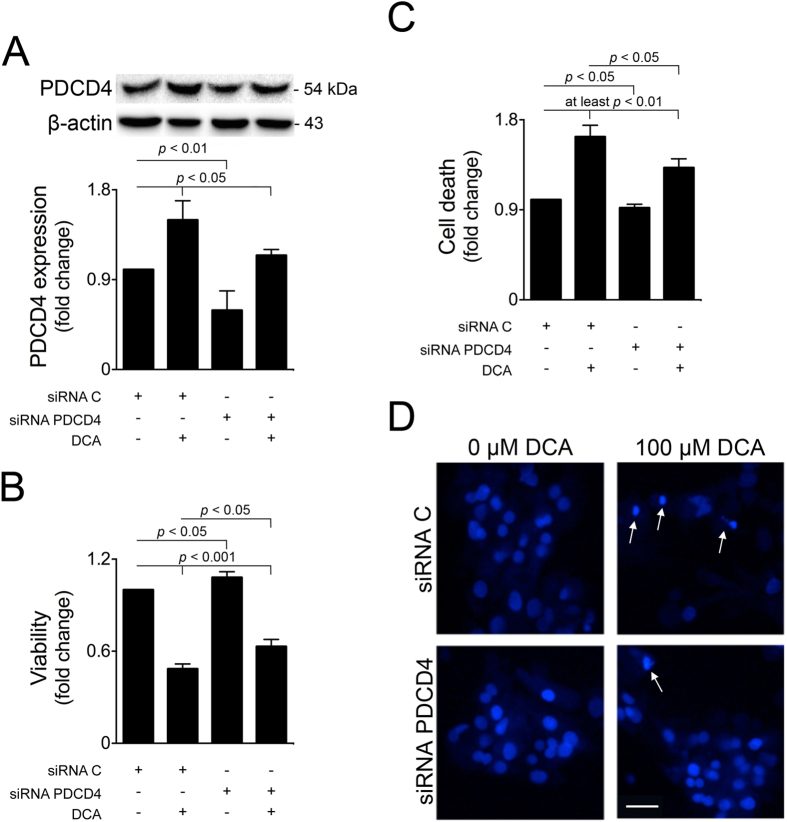
PDCD4 silencing hampers hepatocyte cell death induced by DCA. Primary rat hepatocytes were transfected with a specific siRNA against PDCD4 (siRNA PDCD4) or a control (siRNA C) and treated with 100 μM DCA or no addition for 48 h. (**A**) Immunoblotting of PDCD4 (n = 4). Representative blots are shown. Blots were normalized to endogenous β-actin. (**B**) Cell viability, measured by the ApoTox-Glo^TM^ Triplex assay (n = 4). (**C**) General cell death measured by LDH assay (n = 11). (**D**) Apoptotic cells detected by Hoechst staining. Representative images of control, DCA, PDCD4 silencing and PDCD4 silencing + DCA are shown. Bar, 30 μM. Arrows indicate apoptotic nuclei. Results are expressed as mean ± SEM fold change.

To further evaluate the functional role of PDCD4 during DCA-induced apoptosis of primary rat hepatocytes, we next transfected cells with a specific siRNA against PDCD4, or a control siRNA, in the presence or absence of DCA. Upon silencing, PDCD4 protein levels were significantly inhibited (*p* < 0.01), comparing with control cells (Fig. 3A). PDCD4 inhibition led to a modest but significant increase in cellular viability (Fig. 3B), and decrease in general cell death (Fig. 3C). These results suggest that basal PDCD4 protein levels may not actively contribute to hepatocellular death. However, its activation by DCA is necessary for induction of apoptosis, as PDCD4 silencing significantly impaired the ability of DCA to reduce cellular viability or to increase cell death and apoptosis (Fig. 3,B–D).

Altogether, both miR-21 overexpression and PDCD4 silencing failed to completely abrogate DCA-induced cytotoxicity. This is not surprising, since DCA is thought to engage a series of different apoptotic pathways including, for instance, the miR-34a/SIRT1/p53 pathway^11^. Still, miR-21 and PDCD4 constitute two novel targets modulated by DCA during hepatocyte cell death.

### NF-κB transcriptional activity is inhibited by DCA in primary rat hepatocytes

**Figure 4:**
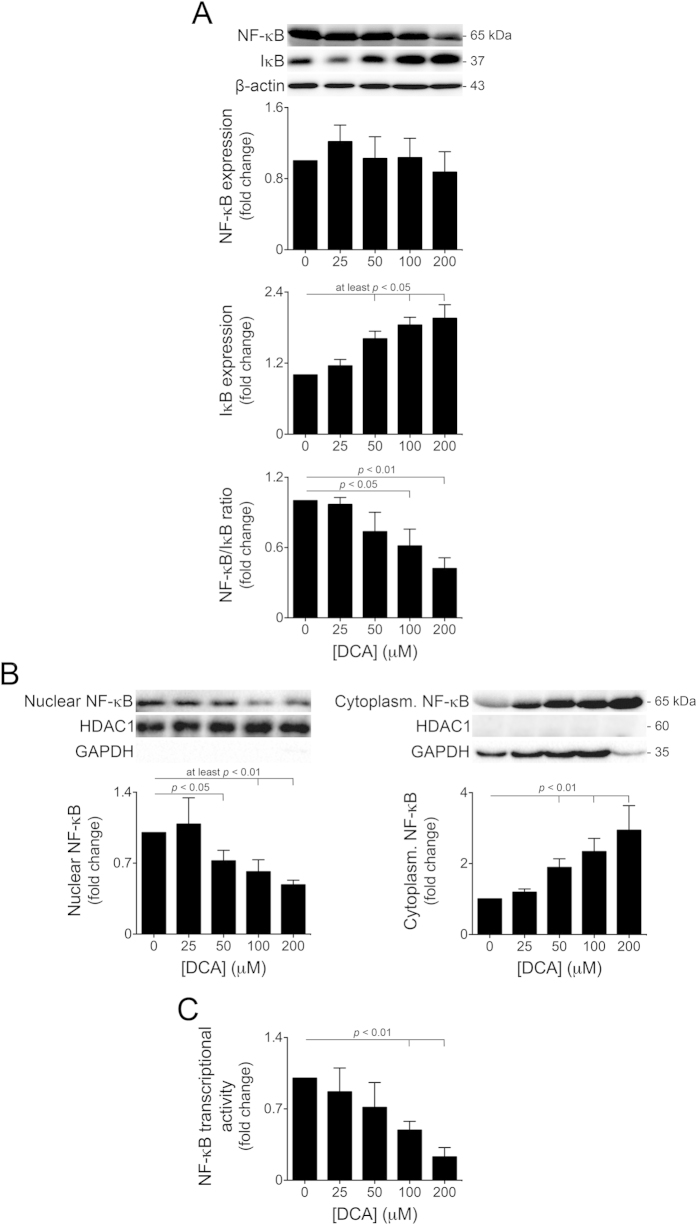
DCA inhibits NF-κB transcriptional activity in primary rat hepatocytes in a dose-dependent manner. Cells were isolated and plated as described in Materials and Methods and treated with 25 to 200 μM DCA or no addition (control) for 24 h. (**A**) Immunoblotting of total NF-κB (top) and IκB (middle); NF-κB/IκB ratio (bottom; n = 5). Representative blots are shown. Blots were normalized to endogenous β-actin. (**B**) Immunoblotting of nuclear NF-κB (left) and cytoplasmatic NF-κB (right; n = 7). HDAC1 and GAPDH were used as nuclear and cytoplasmatic loading controls, respectively, as well as indicators for cross-contamination of protein fractions. Representative blots are shown. (**C**) NF-κB transcriptional activity (n = 5). Cells were transfected with a mixture of an inducible NF-κB responsive construct, encoding the firefly luciferase reporter gene, and a constitutively expressing *Renilla* luciferase construct, as an internal standard control. Results are expressed as mean ± SEM fold change.

NF-κB-mediated survival signalling plays an important role in the liver, and its inhibition has been shown to result in massive hepatocellular apoptosis^33^. Indeed, NF-κB activation protects hepatocytes against glycochenodeoxycholic acid-induced apoptosis^34^. Further, miR-21 was recently described as a direct transcriptional target of NF-κB^26^, in response to genotoxic stress^27^. Consequently, we next investigated whether activation of the miR-21/PDCD4 pro-apoptotic pathway by DCA was dependent on NF-κB expression and/or activity. Primary hepatocytes were incubated with 25 to 200 μM DCA for 24 h. Total NF-κB protein levels were not significantly modulated by DCA (Fig. 4A top). NF-κB is usually located in the cytoplasm, bound to its inhibitor, IκB. Upon activating stimuli, IκB is phosphorylated, ubiquitinated and, consequently, degraded, thus allowing NF-κB nuclear translocation and activation^35^. Our results show that IκB levels were induced by DCA in a dose-dependent manner, for concentrations >25 μM, up to ~2-fold (at least *p* < 0.05) (Fig. 4A middle). As a result, the NF-κB/IκB ratio progressively decreased in cells incubated with increasing concentrations of DCA (Fig. 4A bottom). Our results further show that DCA inhibits NF-κB nuclear translocation, also in a dose-dependent manner (Fig. 4B). To unequivocally establish that DCA inhibits NF-κB activation in primary rat hepatocytes, we next transfected cells with a luciferase construct containing the NF-κB responsive element, in the presence or absence of DCA. DCA decreased NF-κB transcriptional activity in a dose-dependent manner up to ~78% for the 200 μM concentration (*p* < 0.01) (Fig. 4C). Because DCA hampers NF-κB transcriptional activity in a similar pattern to miR-21, these results suggest that DCA-mediated inhibition of miR-21 may constitute an NF-κB downstream event.

### Inhibition of the miR-21/PDCD4 apoptotic pathway by DCA is NF-κB dependent

**Figure 5:**
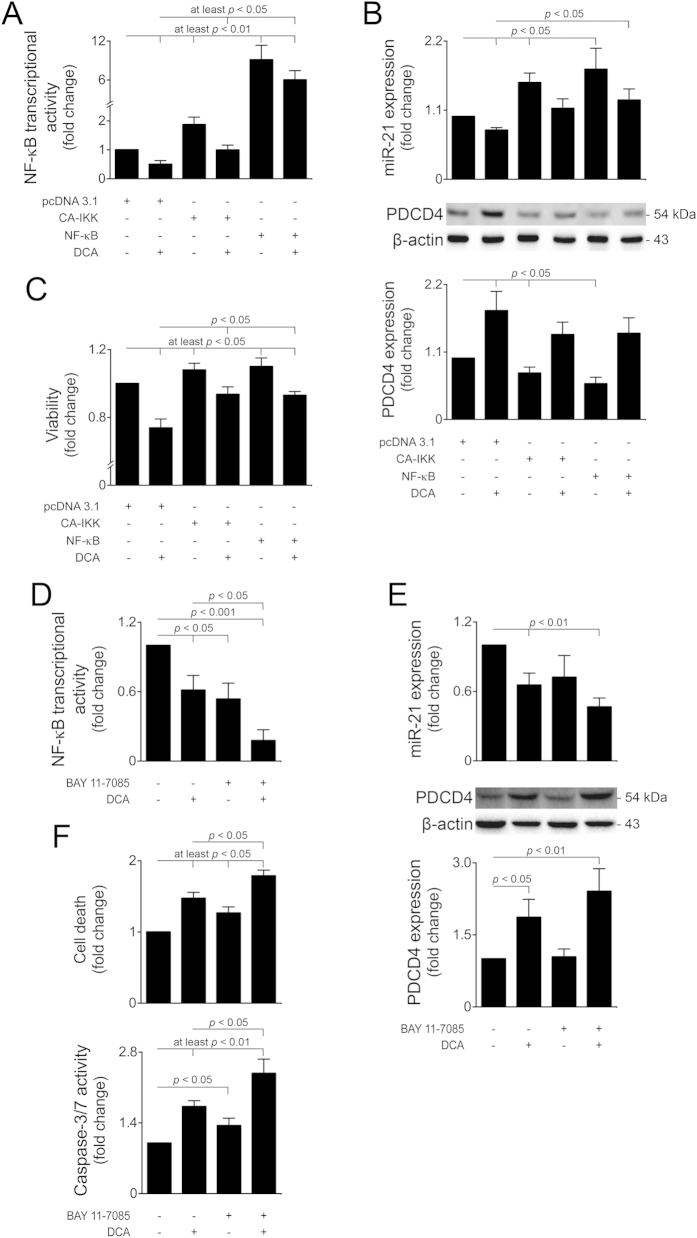
Modulation of the NF-κB pathway halts DCA-modulation of miR-21/PDCD4 and cell viability. Cells were transfected with a plasmid encoding a constitutively active form of IKK (CA-IKK), NF-κB or an empty vector (pcDNA 3.1) or incubated with 10 μM BAY 11-7085 and treated with 100 μM DCA or no addition as described in Materials and Methods. (**A**) NF-κB transcriptional activity (n = 6). Cells were transfected with a mixture of an inducible NF-κB responsive construct, encoding the firefly luciferase reporter gene, and a constitutively expressing *Renilla* luciferase construct, as an internal standard control. (**B**) Real-Time RT-PCR analysis of miR-21 (top; n = 5) and immunoblotting of PDCD4 (bottom; n = 5). Representative blots are shown. Blots were normalized to endogenous β-actin. (**C**) Cell viability, measured by the ApoTox-Glo^TM^ Triplex assay (n = 5). (**D**) NF-κB transcriptional activity (n = 4). (**E**) Real-time RT-PCR analysis of miR-21 (top; n = 6) and immunoblotting of PDCD4 (bottom; n = 8). Representative blots are shown. Blots were normalized to endogenous β-actin. (**F**) Cell death, measured by LDH assay (top; n = 8) and caspase-3/7 activity measured by the ApoTox-Glo^TM^ Triplex assay (bottom; n = 6). Results are expressed as mean ± SEM fold change.

To elucidate the role of NF-κB in our model, we next differently modulated the NF-κB pathway by using a constitutively active form of IKK (CA-IKK), that overactivates the NF-κB pathway by inducing IκB degradation^36^, as well as an NF-κB overexpression construct^37^. Transfection of cells with the CA-IKK and NF-κB plasmids increased NF-κB transcriptional activity by ~2- and 9-fold, respectively (*p* < 0.01), while co-incubation of cells with DCA significantly hampered NF-κB activation (*p* < 0.05) (Fig. 5A). In addition, both CA-IKK and NF-κB overexpression induced miR-21 expression by 40 and 80%, respectively, when compared with controls (*p* < 0.05). Both these effects were almost completely reverted to control levels by DCA (*p* < 0.05) (Fig. 5B top). Additionally, PDCD4 expression profiles displayed an opposite pattern to miR-21 expression levels, further confirming the relevance of this pro-apoptotic target during DCA-dependent modulation of miR-21 *via* NF-κB (Fig. 5B bottom). Finally, activation of the NF-κB pathway by CA-IKK and NF-κB overexpression plasmids resulted in increased cellular viability (at least *p* < 0.05), significantly hampered upon DCA co-incubation (*p* < 0.05) (Fig. 5C).

To further explore to the extent to which NF-κB modulation impacts on DCA-inhibition of the miR-21/PDCD4 pathway, we additionally used a specific chemical inhibitor of NF-κB (BAY 11-7085)^38^. BAY 11-7085 alone reduced NF-κB activity by ~40% (*p* < 0.05), while co-incubation of cells with DCA further potentiated its inhibitory effects (*p* < 0.05) (Fig. 5D). In addition, modulation of the miR-21/PDCA pathway by DCA was also augmented in the presence of BAY 11-7085 (Fig. 5E). Finally, NF-κB inhibition increased general cell death and apoptosis, an effect that was significantly potentiated in the presence of DCA (*p* < 0.05) (Fig. 5F). These effects were further corroborated by transfecting cells with a plasmid coding for a dominant negative IκB (DN-IκB), which inhibits NF-κB activity by increasing the levels of non-phosphorylated IκB (Figure S2). Altogether, these results indicate that the miR-21/PDCD4 pro-apopotic pathway is induced by DCA *via* inhibition of NF-κB activity in primary rat hepatocytes.

### Oxidative stress by DCA, upstream of NF-κB inhibition, downregulates miR-21 and activates caspase-2 and apoptosis

**Figure 6:**
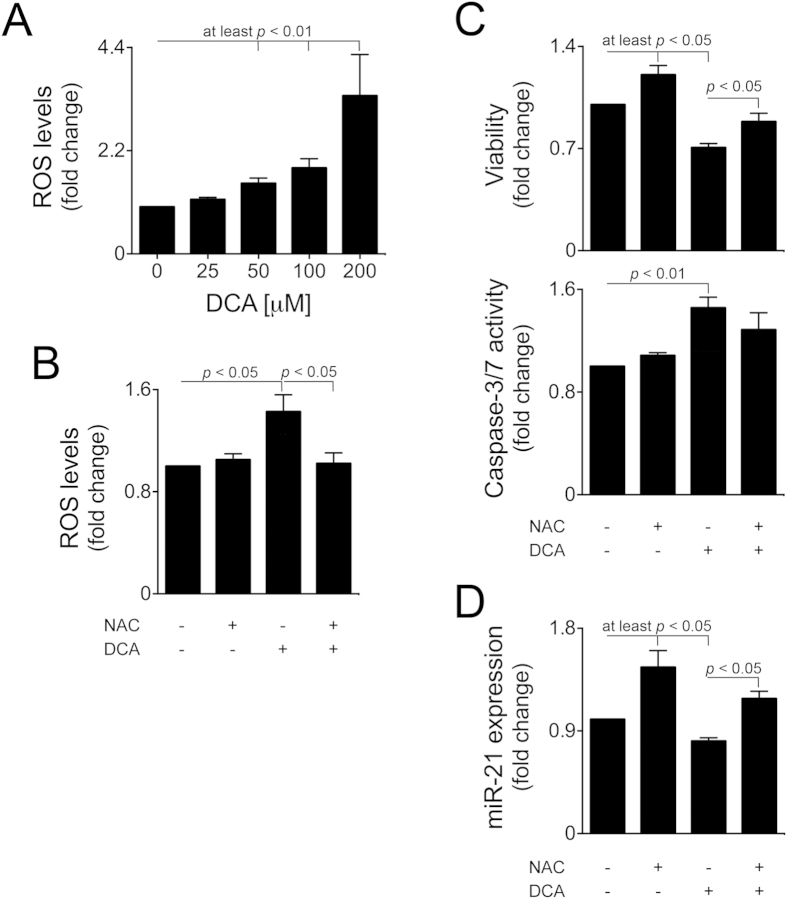
DCA-induced oxidative stress contributes for miR-21 inhibition and apoptosis. Cells were isolated as described in Materials and Methods and treated with 25 to 200 μM DCA or no addition (control) for 16 h (**A**) Dose-dependent modulation of ROS levels by DCA (n = 5). (**B**) ROS levels (n = 3). Cells were pre-treated with 5 mM NAC for 1 h and then incubated with 100 μM DCA for 24 h (or no addition). (**C**) Cell viability (top) and caspase-3/7 activity (bottom) measured by the ApoTox-Glo^TM^ Triplex assay (n = 5). (**D**) Real-Time RT-PCR analysis of miR-21 (n = 5). Results are expressed as mean ± SEM fold change.

DCA induces oxidative stress and DNA damage in hepatocytes^8^,^39^,^40^,^41^. In our model, primary rat hepatocytes incubated with 25–200 μM DCA for 16 h increased ROS production in a dose-dependent manner up to ~3.4-fold (*p* < 0.01) (Fig. 6A). Of note, NF-κB activity is modulated in response to oxidative stress^27^,^42^. To investigate whether triggering of oxidative stress by DCA represents an NF-κB and/or miR-21 upstream mechanism, cells were incubated with NAC, a well-known antioxidant, in the presence or absence of DCA. NAC treatment almost completely abrogated DCA-induced ROS production (*p* < 0.05) (Fig. 6B) and -inhibition of cell viability (*p* < 0.05) (Fig. 6C top), while DCA-induced caspase-3 activity was no longer significant (Fig. 6C bottom). Importantly, DCA-inhibition of miR-21 was also completely abrogated by NAC (Fig. 6D), highlighting the role of oxidative stress during modulation of miR-21 by DCA.

Induction of ROS by DCA likely results in DNA damage^39^. In turn, high levels of genotoxic stress correlate with PIDD-dependent activation of caspase-2. Briefly, upon toxic stimuli, PIDD autoproteolysis is triggered twice, leading to the formation of PIDD-CC, which in turn forms a complex with RAIDD and pro-caspase-2, allowing the activation of the later and thus contributing to apoptosis. Interestingly, PIDD can also activate NF-κB in response to low levels of genotoxic stress. In this scenario, PIDD is processed once (PIDD-C) and activates NF-κB signalling by complexing with receptor interacting protein kinase 1 (RIPK1) and NEMO^43^. As such, DCA-induction of oxidative stress might interfere with the PIDD and caspase-2 networking, thus possibly inhibiting NF-κB and, consequently, miR-21.

**Figure 7:**
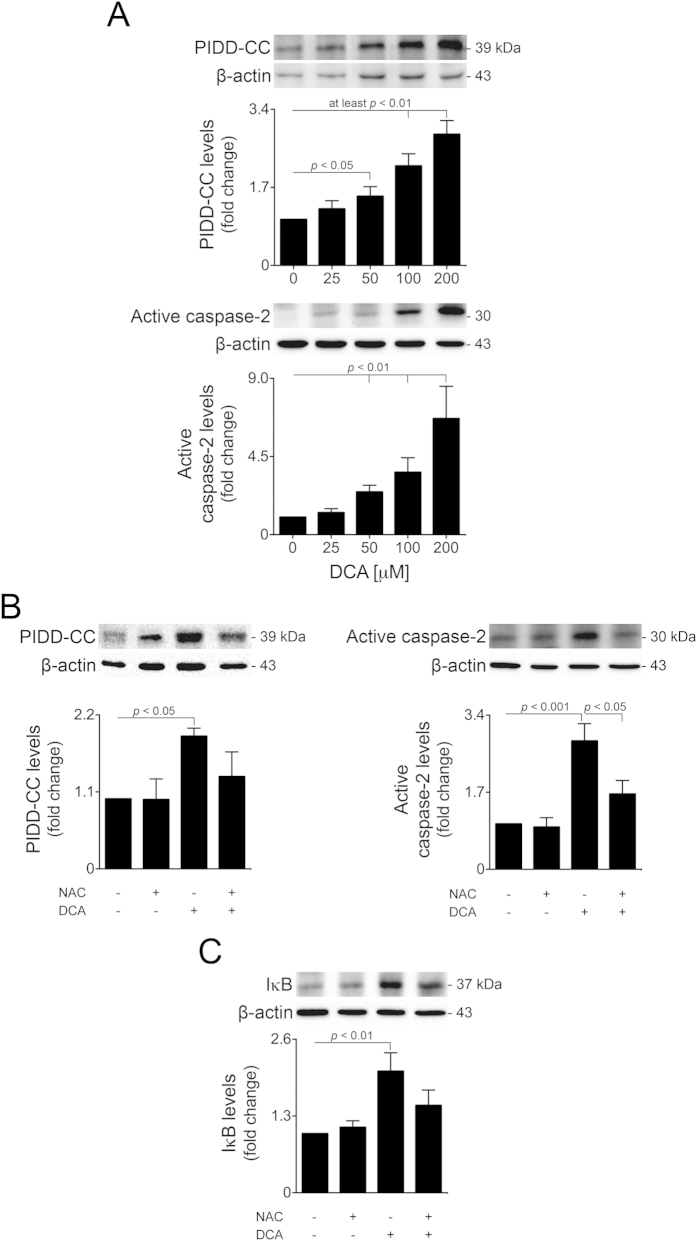
DCA-induced oxidative stress activates PIDD processing and caspase-2 in a dose-dependent manner. Primary rat hepatocytes were isolated as described in Materials and Methods and treated with 25 to 200 μM DCA or no addition (control) for 24 h. (**A**) Immunoblotting of PIDD-CC (top; n = 5) and active caspase-2 (bottom; n = 5). (**B**) Immunoblotting of PIDD-CC (left; n = 5) and active caspase-2 (right; n = 5). (**C**) Immunoblotting of IκB (n = 5). Cells were pre-treated with 5 mM NAC for 1 h and, when indicated, incubated with 100 μM DCA for 24 h. Representative blots are shown. Blots were normalized to endogenous β-actin. Results are expressed as mean ± SEM fold change.

To investigate this hypothesis, we measured PIDD processing and caspase-2 activation in primary rat hepatocytes incubated with 25–200 μM DCA for 24 h. In agreement with the increase in oxidative stress, levels of PIDD-CC and active caspase-2 were augmented by DCA in a dose-dependent manner up to ~2.9- and 8-fold, respectively (at least *p* < 0.05) (Fig. 7A). Of note, caspase-2 knockdown, using a specific siRNA, partially abrogated DCA-induced apoptosis (*p* < 0.05) (Figure S3), suggesting that caspase-2 activation is an important pro-apoptotic event during DCA-induced cell death. Finally, under antioxidant conditions, DCA-dependent PIDD processing was impaired (*p* < 0.05) and activation of caspase-2 significantly reduced (*p* < 0.05) (Fig. 7B). Importantly, DCA-induced IκB levels were also strongly reduced in the presence of NAC, comparing with DCA alone (Fig. 7C). Altogether, these results indicate that DCA-induced oxidative stress is at least partially responsible for inhibition of NF-κB in a PIDD-dependent manner, thus establishing a novel link between genotoxic stress, miR-21 inhibition and cellular toxicity.

### The miR-21/PDCD4 pathway is inhibited in the rat liver *in vivo* by DCA

**Figure 8:**
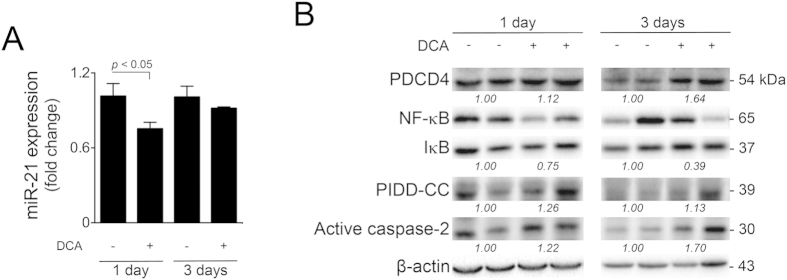
DCA inhibits the NF-κB/miR-21/PDCD4 axis *in vivo*. Wistar male rats (n = 6) were treated with 125 mg/kg/day of DCA by oral gavage for 1 and 3 days before sacrifice. Rats in the control group (n = 6) were administered with water as described in Material and Methods. (**A**) Real-Time RT-PCR analysis of miR-21. Results are expressed as mean ± SEM fold change. (**B**) Representative blots and quantitation, in fold-change from controls, of PDCD4, IκB/NF-κB ratio, PIDD-CC and active caspase-2 from liver of rats treated with DCA for 1 day (left) and 3 days (right). Blots were normalized to endogenous β-actin.

To validate our *in vitro* results and further investigate the physiological relevance of the modulation of the miR-21/PDCD4 pathway by DCA, rats were administered with DCA by oral gavage for 1 to 3 days. As expected, total plasma bile acids, as well as ALT and AST levels increased with time in DCA-treated animals, compared with controls (data not shown). Hepatic miR-21 expression was reduced by  >25% (*p* < 0.05) and ~10% in animals administered with DCA for 1 and 3 days, respectively (Fig. 8A). Concomitantly, liver PDCD4 protein levels were increased by DCA at both 1 and 3 days of treatment (Fig. 8B). In parallel, hepatic total NF-κB protein levels were reduced in DCA-treated animals, while IκB levels increased, leading to a progressive and significant decrease in the NF-κB/IκB ratio throughout time, further reinforcing the role of this transcription factor in modulating the miR-21/PDCD4 pathway. Finally, both PIDD processing and caspase-2 activation were also increased in DCA-treated animals. Curiously, rats administered with DCA for 5 days displayed slightly increased miR-21 expression levels, although positive modulation of PDCD4 was still observed (data not shown), suggesting that sustained DCA administration engages activation of additional pathways of cell death. Overall, our results suggest that PIDDosome activation and consequent inhibition of the NF-κB/miR-21/PDCD4 pathway by DCA occurs both *in vitro* and *in vivo*, providing new mechanistic insights into its role as a putative pathogenic factor for hepatic disorders.

## Discussion

Endogenous bile acids modulate several cell signalling pathways in the liver. Previous studies have show that DCA acts as a potent inducer of hepatocyte cell death *via* both intrinsic and extrinsic apoptotic pathways, and by compromising mitochondrial function^5^,^6^,^9^,^28^. However, the network of apoptotic events upstream of the mitochondria has never been appropriately characterized and few studies have reported the involvement of miRNAs. We recently showed that miR-34a is increased by DCA in a JNK-dependent manner, contributing to liver damage^11^, while miR-21 expression is inhibited in primary rat hepatocytes^25^. In this study, we aimed to elucidate the functional relevance of this inhibition and to evaluate its primary signalling mechanisms. Altogether, our results unravelled NF-κB as a key upstream modulator of the miR-21/PDCD4 pathway. Further, we showed that induction of oxidative stress by DCA leads to impaired NF-κB transcriptional activity, in parallel with caspase-2 activation. As a result, miR-21 expression is decreased, thus allowing PDCD4 levels to rise and facilitate apoptosis. Modulation of the NF-κB/miR-21/PDCD4 pathway by DCA was dose-dependent, for concentrations of DCA above 50 μM. DCA-induced ROS was also increased in a dose-dependent manner, resulting in PIDD processing and caspase-2 activation. Modulation of the miR-21/PDCD4 axis by DCA was also found to be time-dependent, with PDCD4 levels peaking after 24–40 h of incubation, in agreement with the kinetics of miRNA-mediated regulation^44^. The time- and dose-dependent effects of DCA on the NF-κB/miR-21/PDCD4 axis are further consistent with our previous data showing that modulation of miR-34a by DCA is attenuated beyond 52 h of incubation, after which cell death becomes predominantly necrotic^11^.

In the normal adult liver, quiescent hepatocytes express high levels of miR-21^45^. Interestingly, miR-21 expression is further increased upon liver tissue injury or loss^25^,^46^,^47^, suggesting that it may play a role in maintaining liver homeostasis. Our results show that inhibition of miR-21 by DCA results in hepatocyte cell death by apoptosis, in part, as a result of increased PDCD4 levels. PDCD4 appears to contribute to apoptosis by inhibiting AP-1 transactivation and translation, as well as interfering with the cell cycle^48^. In turn, modulation of the JNK1/2 signalling pathway, particularly c-Jun/AP-1, has been shown to contribute for DCA-induced apoptosis^10^,^11^, implying PDCD4 as a novel upstream target.

The miR-21 gene promoter harbours a NF-κB-binding site and several studies have reported that miR-21 is transcriptionally regulated by NF-κB^27^,^49^. In our model, increasing concentrations of DCA progressively decreased both NF-κB/IκB ratio and nuclear NF-κB localization, with a concomitant cytoplasmic accumulation. Of note, a specific phosphorylation of Ser276 in p65 is usually required to promote NF-κB transcriptional activation in the liver^50^. Accordingly, we confirmed our results by specifically measuring NF-κB transcriptional activity, further substantiating the negative modulation of NF-κB by DCA. In addition, modulation of miR-21/PDCD4 by DCA was dependent on this transcriptional repression. In fact, when either CA-IKK or NF-κB was overexpressed, DCA-modulation of the miR-21/PDCD4 pathway was significantly hampered, resulting in lower levels of apoptosis. On the contrary, inhibition of NF-κB by DN-IκB or BAY 11-7085 potentiated the effects of DCA upon miR-21 and apoptosis. Curiously, miR-21 has been described to counteract NF-κB in a negative regulatory loop; during liver regeneration, up-regulation of miR-21 results in decreased Pellino-1 levels, a well-known activator of NF-κB^46^. In our model however, this negative feedback mechanism appears absent or insufficient, likely due to the pronounced and sustained inhibition of NF-κB transcriptional activity by DCA.

Oxidative stress has already been associated with DCA-induced cell death, although the mechanisms are not fully understood. In part, DCA cytotoxicity is thought to be a consequence of mitochondrial dysfunction^8^. In particular, toxic bile acids-induced oxidative stress appear to decrease membrane fluidity, leading to activation of membrane-associated enzymes and ROS^51^. In our model, NAC greatly reduced DCA-induced hepatocyte cell death, while DCA-dependent inhibition of miR-21 was abolished. Hence, oxidative stress appears to be a major triggering factor leading to NF-κB and miR-21 inhibition by DCA. Interestingly, a complex signalling network involving NF-κB, PIDD and caspase-2 has been under scrutiny in the past few years. PIDD is thought to act as a master regulator of this network, deciding between cell death and survival based on the extent of cell damage^52^,^53^. In most cases, PIDD-full length (PIDD-FL) is almost undetectable, substantiating its fast processing; through autoproteolysis, PIDD originates pro-survival PIDD-C, crucial for NF-κB activation, and/or pro-apoptotic PIDD-CC, a fragment sufficient and necessary to promote caspase-2 activation. Additionally, cleavage of these two fragments occurs in a sequential manner, with PIDD-CC arising only from PIDD-C. Reflecting the regulated activity of this second cleavage, high doses of γ-irradiation were shown to induce apoptosis concomitantly with PIDD-CC formation and PIDD-C clearance^43^. In our model, PIDD-CC levels were increased by DCA in a dose-dependent manner, in parallel with activation of caspase-2, in response to increased ROS levels. It is therefore likely that DCA-induced oxidative stress changes the PIDD-C/PIDD-CC ratio, decreasing PIDD-C levels. This would provide a rationale for DCA-inhibition of the NF-κB survival pathway, as a consequence of the PIDD-C, RIPK1 and NEMO platform being disturbed. Indeed, incubation of hepatocytes with NAC inhibited DCA-induced-IκB, -PIDD-CC and -caspase-2. Curiously, a recent report suggested that NF-κB activation is severely compromised in the absence of PIDD, confirming its key role in regulating the NF-κB signalling pathway^54^. Of note, PIDD is also a p53 transcriptional target. As such, it may be additionally targeted by DCA via miR-34a, as we have recently described^11^.

Taken together, our results show that the NF-κB/miR-21 pathway plays a key role during DCA-induced cell death of primary rat hepatocytes and in the rat liver. *In vivo* results have shown that miR-21 expression is down-regulated upon short exposures to DCA, as is the downstream pathway, with concomitant PIDD processing and activation of caspase-2, which contributes to DCA-induced liver damage^55^. This signalling pathway may constitute an important event in early stages of liver apoptosis and/or disease. In fact, DCA was recently shown to contribute to HCC development^3^ and its levels are increased in the serum of NASH patients^2^. In addition, DCA is one of the bile acid species that more dramatically increases in experimental steatohepatitis, likely potentiating liver disease^56^. Interestingly, we have recently shown that both p53 and caspase-2 activation are increased in patients with different stages of NASH^55^,^57^ and that DCA induces p53 activation in the rat liver^11^. Altogether, the increased levels of DCA in NASH patients likely contribute to sequential oxidative stress, p53 transcription, PIDD processing and caspase-2 activation, culminating, at least in part, in NF-κB/miR-21/PDCD4-depdendent cell death and toxicity. Our results show that inhibition of NF-κB by DCA constitutes a key cytotoxic signalling event; NF-κB was shown to protect the liver against fibrosis by preventing apoptosis^58^ and also prevents bile acid-induced apoptosis in cholestatic liver injury^34^.

In this work, key concepts regarding the cytotoxic mechanisms induced by DCA were unravelled; in particular, we showed that DCA induces apoptosis in primary rat hepatocytes by targeting NF-κB/miR-21. Because bile acids are rising as key pathogenic factors in liver diseases, particularly NAFLD, our results also highlight the potential of these novel targets to treat liver patients.

## Methods

### Cell culture and treatments

Primary rat hepatocytes were isolated from male rats (100 to 150 g) by collagenase perfusion as previously described^11^. After isolation, hepatocytes were resuspended in complete Williams E medium (Sigma-Aldrich Co., St. Louis, MO, USA) and plated on BD Primaria™ culture dishes (BD Biosciences, San Jose, CA, USA) at 5 × 10^4^ cells/cm^2^. Cells were kept at 37 °C in a humidified atmosphere of 5% CO_2_ for 4–6 h to allow attachment. Plates were then washed with phosphate buffered saline (PBS) 1× in order to remove dead cells and incubated in Williams E medium supplemented with 25 to 200 μM DCA (Sigma-Aldrich Co.) or no addition (control) for 24 h. Primary rat hepatocytes were processed for total RNA and protein isolation, cell viability, cytotoxicity and caspase activity assays and Hoechst staining. Nuclear and cytoplasmic fractions were also obtained. Alternatively, for time-dependent experiments, cells were incubated with 100 μM DCA or no addition (control) for 4, 16, 24, 40 and 48 h. Cells were harvested and processed for total protein and RNA extractions.

### PDCD4 reporter assays

To ascertain miR-21-dependent modulation of PDCD4 by DCA, a reporter plasmid driven by the SV40 basal promoter, harbouring either a wild-type (Luc-PDCD4 Wt 3′ untranslated region (UTR)) or a mutated (Luc-PDCD4 Mut 3′ UTR) miR-21 target sequence within the 3′ UTR of PDCD4, upstream of the Firefly luciferase gene, was used^32^. At the time of plating, primary rat hepatocytes were transfected with 200 ng of the luciferase reporter construct, or a reporter control, using Lipofectamine^TM^ 2000 (Life Technologies, Carlsbad, CA, USA). Cells were also co-transfected with 50 ng of pRL-SV40 (Promega Corp., Madison, WI, USA). *Renilla* luciferase activity was used for transfection normalization purposes. Six hours after transfection, plates were washed with PBS 1X and incubated with 25 to 200 μM DCA for 24 h. Reporter assays were performed using the Dual-Luciferase® Reporter Assay System (Promega Corp.), according to the manufacture’s specifications.

### miR-21 and PDCD4 functional modulation

For functional analyses, primary rat hepatocytes were transfected at the moment of plating with 100 pM of a specific miR-21 precursor (pre-miR-21; AM17100) (Ambion, Life Technologies) or with a pre-miR negative control (pre-miR-C; AM17110), using Lipofectamine 2000™. After 6 h, cells were incubated with 100 μM DCA or no addition (control). Hepatocytes were harvested 24 h after incubation with DCA and processed for total protein and RNA extraction, as well as cell viability and caspase activity assays. To ascertain the relevance of PDCD4 in DCA-induced cell death, primary rat hepatocytes were transfected with a specific Silencer® Select short interference RNA (siRNA; si-PDCD4) designed to knock down *PDCD4* gene expression in rats (#4390771; Ambion, Life Technologies). A silencer negative-control siRNA that does not lead to the degradation of any known cellular mRNA was used as control (#4390844; Ambion, Life Technologies). Primary rat hepatocytes were transfected with siRNA at a final concentration of 100 pM using Lipofectamine 2000™ at platting and incubated with 100 μM DCA or no addition (control) 6 h after transfection. Hepatocytes were harvested 48 h after DCA incubation and processed for total protein extraction and cell viability and cytotoxicity assays, as well as Hoechst staining.

### NF-κB transcriptional activity assays

The effects of DCA in NF-κB transcriptional activity were assessed using the Cignal NF-κB Pathway Reporter Assay Kit (CCS-013L; QIAGEN, Chatsworth, CA, USA), according to the manufacturer’s specifications. Briefly, it comprises an inducible NF-κB responsive construct, which encodes the firefly luciferase reporter gene, and a constitutively expressing *Renilla* luciferase construct. Primary rat hepatocytes were transfected when plated using Lipofectamine 2000™ with 100 ng of each plasmid (reporter or negative control plasmid). Cells were washed with PBS 1 × 6 h after transfection and incubated with 25 to 200 μM DCA. After 24 h, cells were processed for the Dual-Luciferase® Reporter Assay System.

To evaluate the effects of NF-κB activity upon the miR-21/PDCD4 pathway, 2 μg of a plasmid encoding NF-κB, a plasmid encoding the constitutively active form of IKK or a dominant negative IκB plasmid (21966, 11105 and 12329, respectively; Addgene) were transfected into primary rat hepatocytes at plating. Cells were incubated with 100 μM DCA or no addition (control) 6 h after transfection and processed for RNA and total protein, as well as for cell viability assays, 48 h after incubation with DCA. In parallel, to evaluate the different NF-κB activation status achieved with these plasmids, primary rat hepatocytes were co-transfected at plating with 400 ng of each plasmid along with 100 ng of a plasmid containing an inducible NF-κB responsive element upstream of the firefly gene, as described above, or a negative control construct. Cells were washed with PBS 1 × 6 h after transfection and incubated with 100 μM DCA or no addition (control) for 48 h. Cells were then harvested and processed for the Dual-Luciferase® Reporter Assay System. Finally, in parallel experiments, 10 μM of a chemical NF-κB inhibitor (BAY 11-7085; Sigma-Aldrich Co.) was co-incubated with 100 μM DCA in primary rat hepatocytes for 24 h.

### Inhibition of oxidative stress and caspase-2

To assess the role of ROS production/genotoxic stress in our system, 5 mM of a well-established antioxidant, N-acetyl-L-cysteine (NAC; Sigma-Aldrich Corp.) was used to inhibit DCA-induced oxidative stress. Briefly, after plating, cells were washed with PBS 1X and incubated with NAC for 1 h. Cells were then incubated with 100 μM DCA for 24 h before harvesting for total protein and RNA extraction and for viability and caspase activity assays. To confirm inhibition of ROS by NAC, cells were incubated with DCA for 8 h after NAC addition and then processed for total ROS measurement. In order to ascertain the relevance of caspase-2 activation in DCA-induced apoptosis, primary rat hepatocytes were transfected with 50 pM of a Silencer® Select caspase-2 siRNA, or a Silencer® negative-control siRNA (#4390771 and #4390844, respectively; Ambion, Life Technologies) upon plating and incubated with 100 μM DCA or no addition (control). Cells were harvested 24 h after DCA incubation and processed for total protein extraction and cell viability and caspase assays.

### Animals and DCA treatment

Male Wistar rats (Harlan Laboratories Models, S.L., Spain) weighing ~150 g were maintained on a 12-h light-dark cycle and fed standard laboratory chow *ad libitum*. Water (*n* = 3) or DCA (*n* = 3) at a dose of 125 mg/kg of body weight, given twice a day, was administered by oral gavage for 1 and 3 consecutive days. Body weight was monitored every day. On each time point, animals were weighed and liver perfusion, with cold PBS 1X, was performed after sacrifice. The liver was quickly removed, rinsed in normal saline, flash-frozen in liquid nitrogen and stored at −80 °C. All experiments involving animals were performed by an investigator accredited for directing animal experiments (FELASA level C), in agreement with the Public Health Service (PHS) Policy on Human Care and Use of Laboratory Animals, incorporated in the Institute for Laboratory Animal Research (ILAR) Guide for Care and Use of Laboratory Animals. Experiments received prior approval from the Portuguese National Authority for Animal Health (DGAV).

### Real-Time RT-PCR

Total RNA was extracted using the TRIzol® reagent according to the manufacturer’s instructions (Life Technologies). Real-Time RT-PCR was performed in an Applied Biosystems 7300 system (Life Technologies) to quantitate the expression of miR-21. U6 small nuclear RNA (snRNA) was used as the normalization control. The relative amount of miR-21 was determined by the threshold cycle (2^−ΔΔ*CT*^) method, where ΔΔC_T_ = (C_TmiR-21_ − C_TU6_) sample−(C_TmiR-21_ − C_TU6_) calibrator.

### Immunoblotting

Seventy or thirty micrograms of total protein extracts or nuclear/cytoplasmic extracts, respectively, were separated by 10% sodium dodecyl sulphate-polyacrilamide gel electrophoresis (SDS-PAGE). Upon transference onto a nitrocellulose membranes and blocking with 5% milk solution, membranes were probed overnight at 4 °C with primary rabbit polyclonal antibodies against IκB (sc-371), NF-κB p65 (sc-372) and PIDD (sc-67032) (Santa Cruz Biotechnology, Santa Cruz, CA, USA), primary rabbit polyclonal antibody against active caspase-2 (ab2251; Abcam PLC, Cambridge, United Kingdom), primary goat polyclonal antibody against PDCD4 (sc-27123; Santa Cruz Biotechnology) or primary mouse monoclonal antibodies against β-actin (A5441; Sigma-Aldrich Co.), HDAC1 (05–100; Upstate Biotechonology, Lake Placid, NY, USA), GAPDH (sc-32233; Santa Cruz Biotechnology) and, finally, with secondary antibodies conjugated with horseradish peroxidase (Bio-Rad Laboratories, Hercules, CA, USA) for 3 h at room temperature. Membranes were processed for protein detection using Super Signal substrate (Pierce, Rockford, IL, USA). Protein concentrations were determined using the Bio-Rad protein assay kit (Bio-Rad Laboratories) according to the manufacturer’s specifications.

### Hoechst staining

After cell treatments, culture medium was gently removed to prevent cells detachment. Primary rat hepatocytes were then fixed with 4% paraformaldehyde in PBS, pH 7.4, for 20 min at room temperature, washed with PBS, and incubated with Hoechst dye 33258 (Sigma-Aldrich Corp.) at 5 μg/mL in PBS for 10 min, protected from light. Cells were finally washed with PBS and mounted with coverslips using PBS/glycerol (3:1). Nuclear morphology was visualized by fluorescence using an Axioskop fluorescence microscope (Carl Zeiss GmbH, Jena, Germany). Blue-fluorescent nuclei were blindly scored according to the condensation and staining characteristics of chromatin. While normal nuclei showed non-condenses chromatin disperse over the entire nucleus, apoptotic nuclei were recognised by condensed chromatin, contiguous to the nuclear membrane, as well as by nuclear fragmentation. Five random microscopic fields per sample containing approximately 100 nuclei were analysed.

### Cell viability, cytotoxicity and caspase activity measurements

The ApoTox-Glo^TM^ Triplex assay was used according to the manufacturer’s specifications (Promega Corp.), allowing for measurement of viability, cytotoxicity and caspase-3/7 activity in the same sample well. Alternatively, cells were incubated with the Caspase-Glo® 3/7 reagent. (Promega Corp.). General cell death was assessed by measuring lactate dehydrogenase (LDH) activity. Briefly, cell culture supernatants were combined in microplates with lactate (substrate), tetrazolium salt (coloring solution), and NAD (cofactor), previously mixed equitably according to the protocol (Roche Applied Science, Indianapolis, IN, USA). Plates were protected from light and incubated for at least 5 min at room temperature. Finally, absorbance was measured at 490 nm, with 620 nm as reference, using a Bio-Rad model 680 microplate reader (Bio-Rad Laboratories).

### Total ROS measurement

Primary rat hepatocytes were incubated with 25 to 200 μM DCA or no addition (control) 4 h after plating. 16 h after, cells were incubated for 30 min at 37 °C with 10 μM 2′,7′-dichlorodihydrofluorescein diacetate (H_2_DCFDA; Sigma-Aldrich Co.), a cell-permeant nonfluorescent molecule that is oxidized by ROS, resulting in the accumulation of a fluorescent compound (dichlorofluorescein). Cells were washed with PBS 1X and the emission of green fluorescence measured using the GloMax-Multi^+^ Detection System (Promega Corp.). Total protein levels were used for normalization purposes. For measuring ROS levels upon NAC incubation, cells were pre-treated with NAC for 1 h and then incubated with DCA for additional 8 h.

### Densitometry and statistical analyses

The relative intensities of protein bands were analysed using ImageLab Version 5.1 densitometric analysis program (Bio-Rad Laboratories). Statistical analysis was performed using GraphPad Prism version 5.0 (GraphPad Software, San Diego, CA). One-way analysis of variance (ANOVA) test was used for comparisons when more than two groups were analysed and Student’s *t*-test when two groups were analysed. ANOVA was performed using the Dunnet´s (dose-dependent and time course experiments) or Tukey’s post tests. Values of *p* < 0.05 were considered significant.

## Additional Information

**How to cite this article**: Rodrigues, P. *et al.* Inhibition of NF-κB by deoxycholic acid induces miR-21/PDCD4-dependent hepatocellular apoptosis. *Sci. Rep.* **5**, 17528; doi: 10.1038/srep17528 (2015).

## Supplementary Material

Supplementary Information
