# Corrigendum: Inhibition of NF-κB by deoxycholic acid induces miR-21/PDCD4-dependent hepatocellular apoptosis

**DOI:** 10.1038/srep27828

**Published:** 2016-06-24

**Authors:** Pedro M. Rodrigues, Marta B. Afonso, André L. Simão, Pedro M. Borralho, Cecília M. P. Rodrigues, Rui E. Castro

Scientific Reports 5: Article number: 1752810.1038/srep17528; published online: 12012015; updated: 06242016

The original version of this Article contained an error in the title of the paper, where the word “hepatocellular” was incorrectly given as “hepatocelular”. This has now been corrected in the PDF and HTML versions of the Article.

