# Supplementary material for: Inhibition of NF-κB by deoxycholic acid induces miR-21/PDCD4-dependent hepatocellular apoptosis
Source: Sci Rep. 2015 Dec 1;5:17528. doi: 10.1038/srep17528 (PMC4664913; doi:10.1038/srep17528)
Supplement: Supplementary Information [file srep17528-s1.pdf]

Inhibition of NF-κB by deoxycholic acid induces miR-21/PDCD4-dependent hepatocelular apoptosis

Pedro Miguel Rodrigues[1], Marta Bento Afonso[1], André Lopes Simão[1], Pedro Miguel Borralho[1,2], Cecília Maria Pereira Rodrigues[1,2*], Rui Eduardo Castro[1,2*]

[1]Research Institute for Medicines (iMed.ULisboa), [2]Department of Biochemistry and Human Biology, Faculty of Pharmacy, Universidade de Lisboa, 1649-003 Lisboa, Portugal.

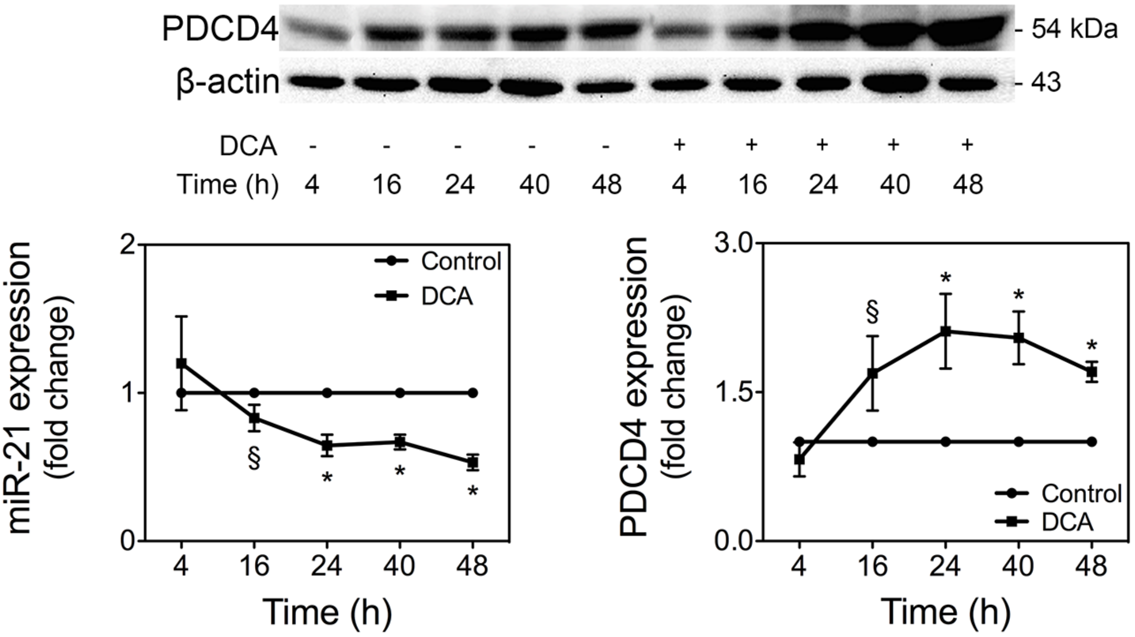

Figure S1 | DCA inhibits the miR-21/PDCD4 axis in a time-dependent manner. Cells were isolated as described in Materials and Methods and treated with 100 μM DCA or no addition (control) for 4, 16, 24, 40 and 48h. Real-Time RT-PCR analysis of miR-21 (left; n=6) and immunoblotting of PDCD4 (right; n=6). Representative blots are shown. Blots were normalized to endogenous β-actin. Results are expressed as mean ± SEM fold change. $^{§}p < 0.05$ and $^{*}p < 0.01$ from control.

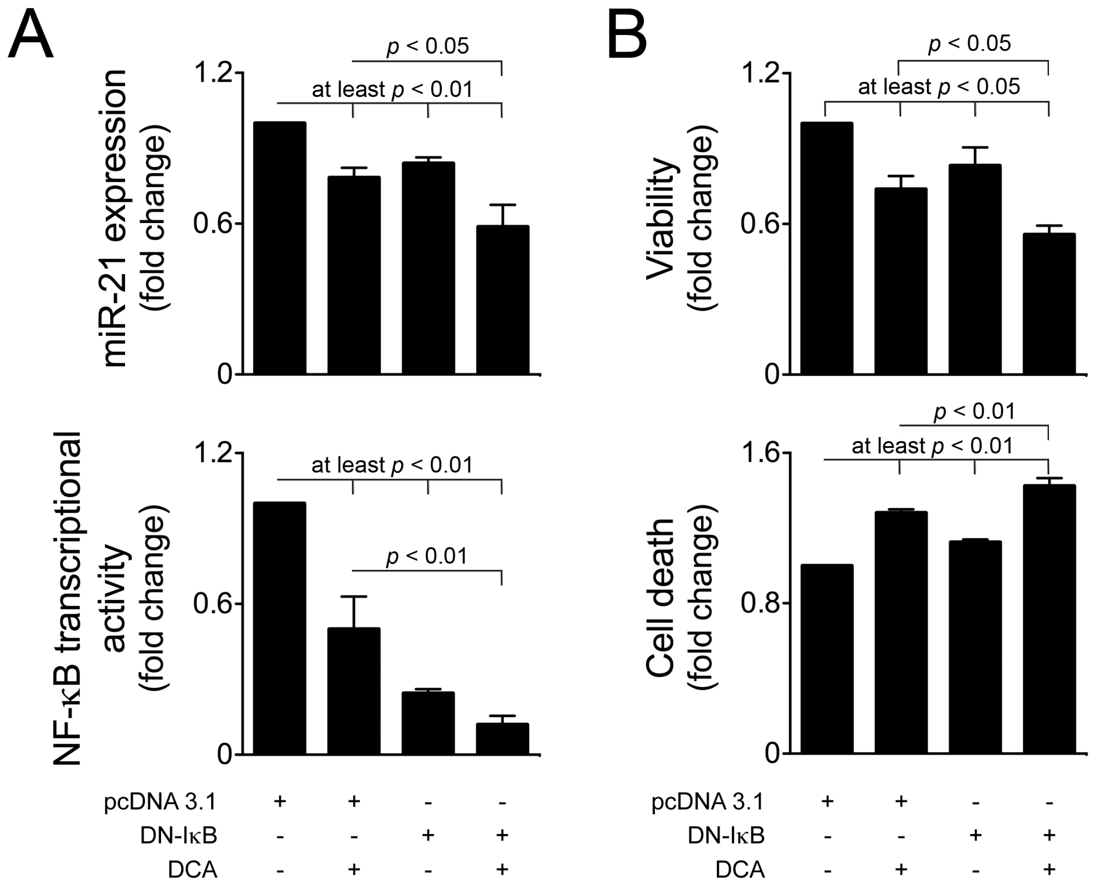

Figure S2 | Constitutive inhibition of the NF-κB pathway potentiates the effects of DCA in modulating miR-21 and cell death. Cells were transfected with a plasmid encoding a dominant negative form of IκB (DN-IκB) or an empty vector (pcDNA 3.1) and treated with 100 μM DCA or no addition for 48 h, as described in Materials in Methods. (**A**) Real-Time RT-PCR analysis of miR-21 (top; n=5) and NF-κB transcriptional activity (bottom; n=4). Cells were transfected with a mixture of an inducible NF-κB responsive construct, encoding the firefly luciferase reporter gene, and a constitutively expressing *Renilla* luciferase construct, used as an internal standard control. (**B**) Cell viability, measured by the ApoTox-Glo™ Triplex assay (top; n=5) and cell death, measured by LDH assay (bottom; n=5). Results are expressed as mean ± SEM fold change.

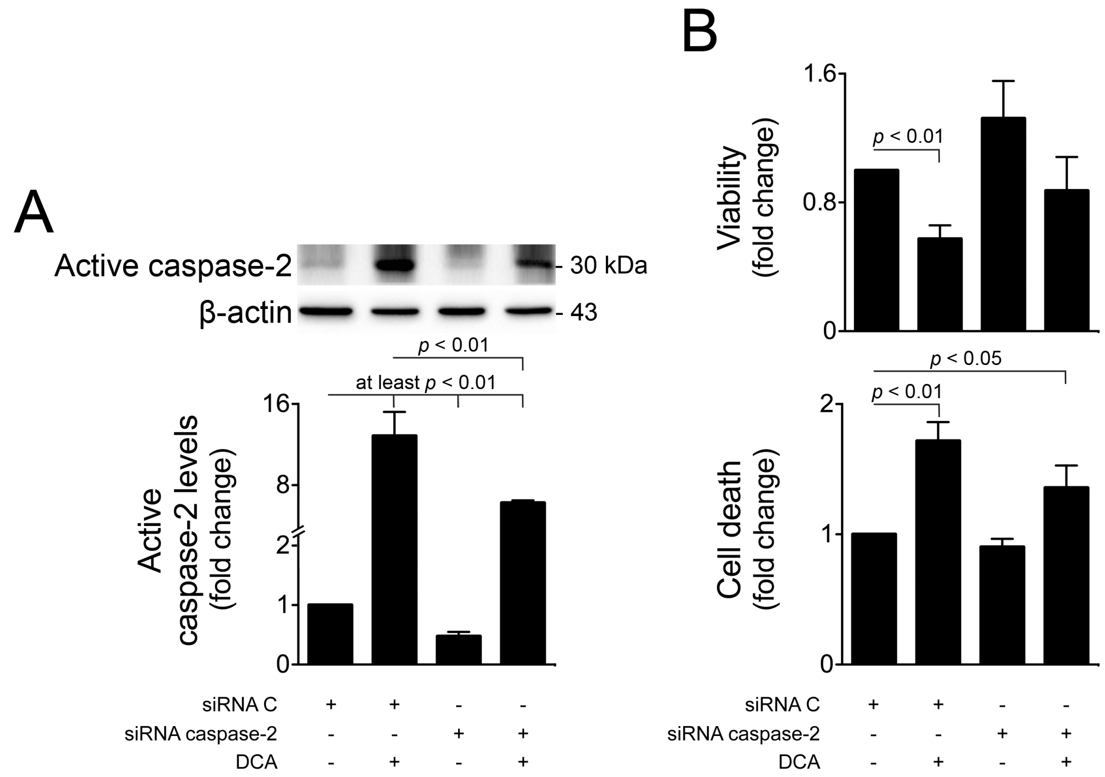

Figure S3 | Caspase-2 activation contributes to DCA-induced cell death. Primary rat hepatocytes were transfected with a specific siRNA against caspase-2 (siRNA caspase-2) or a control (siRNA C) and treated with 100 μM DCA or no addition for 24 h. (**A**) Immunoblotting of active caspase-2 (n=5). Representative blots are shown. Blots were normalized to endogenous β-actin. (**B**) Cell viability measured by the ApoTox-Glo^{TM} Triplex assay (top; n=4) and cell death measured by LDH assay (bottom; n=4). Results are expressed as mean ± SEM fold change.
